# Molecular Identification of *Palmistichus elaeisis*, *Tetrastichus howardi*, *Trichospilus diatraeae* and *Trichogramma pretiosum* (Hymenoptera: Chalcidoidea)—Important Biocontrol Agents

**DOI:** 10.3390/insects17040395

**Published:** 2026-04-05

**Authors:** Izabella de Lima Palombo, Fabricio Fagundes Pereira, André Pessoa da Costa, Patrik Luiz Pastori, Alex Polatto Carvalho, Andrea Renata da Silva Romero, André Vieira do Nascimento, Ana Maria Perez Obrien, Patricia Iana Schmidt, Carlos Reinier Garcia Cardoso, Marcelo Teixeira Tavares

**Affiliations:** 1 Faculdade de Ciências Biológicas e Ambientais, Universidade Federal da Grande Dourados, Rodovia Dourados/Itahum, Km 12, Dourados 79804-970, MS, Brazil; izabella.palombo060@academico.ufgd.edu.br (I.d.L.P.); andrecosta1970@hotmail.com (A.P.d.C.); 2 Faculdade de Ciências Agrárias, Universidade Federal da Grande Dourados, Rodovia Dourados/Itahum, Km 12, Dourados 79804-970, MS, Brazil; patrikpastori@ufgd.edu.br (P.L.P.); alexpolatto@hotmail.com (A.P.C.); 3 Empresa Agropartners Consulting, Centro, Araçatuba 16010-220, SP, Brazil; andrea@agropartners.com.br (A.R.d.S.R.); andrevn@agropartners.com.br (A.V.d.N.); anamaria@agropartners.com.br (A.M.P.O.); patricia@agropartners.com.br (P.I.S.); 4 Empresa Sistêmica Kovê Ltda., Rodovia Dourados/Itahum, Km 12, Dourados 79804-970, MS, Brazil; cr.garcia.cardoso@gmail.com; 5 Centro de Ciências Humanas e Naturais, Departamento de Ciências Biológicas, Universidade Federal do Espírito Santo, Vitória 29043-900, ES, Brazil; tavares.mt@gmail.com

**Keywords:** biological control, parasitoids, genomic sequencing

## Abstract

Parasitoid wasps play a fundamental role in the biological control of pests. However, their morphological identification may be limited due to their small size and the high morphological similarity between species. Our objective was to identify specific genomic variants of the target species *Palmistichus elaeisis* Delvare & LaSalle, 1993, *Tetrastichus howardi* (Olliff, 1893), *Trichospilus diatraeae* Cherian & Margabandhu, 1942, and *Trichogramma pretiosum* Riley, 1879, (Hymenoptera: Chalcidoidea) by whole-genomic sequencing. Parasitoids were collected from their hosts and established in the laboratory after adult emergence. A sample of each parasitoid was sent to the Departamento de Ciências Biológicas at Universidade Federal do Espírito Santo (UFES) and “Oscar Monte” Entomophagous Insect Collection for morphological identification. Subsequently, samples composed of 20 individuals were preserved in absolute ethanol for DNA extraction. The DNA was extracted, quantified and sequenced on the Illumina Novaseq 6000 platform. Bioinformatic tools were used for quality control, detection of specific genomic variants, principal component analysis (PCA), and support vector machine (SVM). Genomic sequencing generated high-quality data for the analyzed parasitoids, allowing the identification of four specific variants for *P. elaeisis*, two for *Te. howardi*, four for *Ts. diatraeae* and five for *Tg. pretiosum*. These results provide a precise molecular tool for distinguishing parasitoids used in biological control programs.

## Simple Summary

Parasitoid wasps play a fundamental role in the biological control of pests. However, their morphological identification can be limited due to the high morphological similarity between species. Our objective was to identify specific genomic variants of the target species *Palmistichus elaeisis*, *Tetrastichus howardi*, *Trichospilus diatraeae* and *Trichogramma pretiosum* by whole-genomic sequencing. The parasitoids were collected from their hosts and reared in the laboratory. Subsequently, samples composed of adult specimens were preserved in absolute ethanol for morphological and molecular identification. Genomic sequencing generated high-quality data for the four parasitoid species analyzed, allowing for the consistent identification of specific genomic variants. These results provide a precise molecular tool for distinguishing parasitoids used in biological control programs.

## 1. Introduction

Parasitoid wasps act as natural enemies of a wide range of arthropods, being widely used as biological control agents to regulate insects that cause economic damage in agricultural and forestry systems [1]. In recent years, several parasitoid species have been identified and recorded as biological products in agricultural crops in Brazil [2,3]. Examples include the gregarious endoparasitoids *Palmistichus elaeisis* Delvare & LaSalle, 1993, *Tetrastichus howardi* (Olliff, 1893), *Trichospilus diatraeae* Cherian & Margabandhu, 1942 (Hymenoptera: Eulophidae), and the egg parasitoid *Trichogramma pretiosum* Riley, 1879 (Hymenoptera: Trichogrammatidae). These species are used to control various lepidopteran species, especially in integrated pest management programs [2].

Accurate identification of these species is fundamental for the success of biological control programs, since incorrect identification can compromise the efficiency of parasitoid releases and the interpretation of ecological interactions. Traditionally, insect identification is based on the description and classification of species according to observable external and internal characteristics [4]. However, in groups of parasitoid wasps, this approach has some limitations due to the great diversity of species, their minuscule body size, and the high demand for specialized taxonomic expertise [5].

The challenges of species identification are evident in the families Eulophidae and Trichogrammatidae due to the number of morphologically similar species. The family Eulophidae comprises approximately 6000 described species distributed across 347 genera and exhibits high morphological and genetic diversity [6,7]. Species such as *P. elaeisis*, *Te. howardi*, and *Ts. diatraeae* share morphological and behavioral similarities in host selection, frequently parasitizing the eucalyptus brown looper *Thyrinteina arnobia* (Stoll, 1782) (Lepidoptera: Geometridae) and the sugarcane borer *Diatraea saccharalis* (Fabricius, 1794) (Lepidoptera: Crambidae) thereby contributing to insect population balance [2,8,9]. Similarly, Trichogrammatidae has a worldwide distribution and is represented by 89 genera and more than 800 described species of egg parasitoid wasps [10]. Among these, *Tg. pretiosum* can be easily confused with other species of the genus, since its identification depends on the combination of morphological characteristics of the male genitalia, type of antennae, wings and body pigmentation [11,12]. This is one of the most utilized species for the biological control of tomato leafminer *Tuta absoluta* (Meyrick, 1917) (Lepidoptera: Gelechiidae), in tomato crops in Brazil [13].

Due to the economic and ecological importance of these parasitoid insects, the correct identification of species is fundamental for the success of biological control programs. In this context, molecular approaches have emerged as complementary tools for the rapid and accurate identification and differentiation of species [14]. More consistent genetic information is available for *Tg. pretiosum* [15,16], whereas *P. elaeisis*, *Te. howardi*, and *Ts. diatraeae* remain underrepresented in molecular studies.

To date, available molecular data for these species are restricted to specific genetic markers, such as mitochondrial genes or ribosomal regions. Although useful, these markers may have limited resolution for distinguishing closely related species [17]. Genomic variants correspond to differences in the deoxyribonucleic acid (DNA) sequence among individuals or species and represent an important source of genetic information for comparative genomic analyses. These variants include single-nucleotide polymorphisms (SNPs), insertions and deletions (indels), and other structural differences distributed across the genome, which can provide higher resolution for distinguishing closely related taxa [18].

The complete mitochondrial genome of *Te. howardi* has been sequenced, providing insights into its genetic composition and phylogenetic relationships within the family Eulophidae [19]. Similarly, the 28S rDNA region of *Ts. diatraeae* has been sequenced, with phylogenetic analyses corroborating its placement within this family [20].

In contrast, whole-genome sequencing (WGS) is a flexible high-throughput technology that enables the generation of whole-genome data and the construction of genomic libraries, allowing the identification of unique regions distributed throughout the genome and providing a more robust alternative for the molecular identification of these parasitoids [21]. Furthermore, it is possible to analyze genomic variants that correspond to differences in DNA sequences within species. Among these, the analysis of single-nucleotide polymorphisms (SNPs) is a powerful tool for genetic studies, evolutionary processes, and comparative analyses between taxa. SNPs are widely used due to their abundance in genomes and their functional relevance [18].

In this study, we employed Illumina NovaSeq 6000 sequencing to generate complete genomic data and identify polymorphic regions to differentiate species-specific genomic variants, thereby ensuring accurate identification for biological control programs [22]. Therefore, our objective was to identify specific genomic variants of the target species *P. elaeisis*, *Te. howardi*, *Ts. diatraeae* and *Tg. pretiosum* by whole-genomic sequencing.

## 2. Materials and Methods

The work was developed at the Laboratory of Biological Control of Insects (LECOBIOL) of the Federal University of Grande Dourados in the city of Dourados, state of Mato Grosso do Sul, Brazil, in partnership with Agropartners Consulting Company of Araçatuba, São Paulo, Brazil.

### 2.1. Insect Collection

Parasitoids were collected from their hosts, and a rearing was established at LECOBIOL after adult emergence. The species *P. elaeisis* and *Ts. diatraeae* were obtained from pupae of *T. arnobia* in eucalyptus crops [23,24], while *Te. howardi* was collected in pupae of *D. saccharalis* in sugarcane stalks [25]. A sample of each pupal parasitoid was sent to the Departamento de Ciências Biológicas at Universidade Federal do Espírito Santo (UFES) for morphological identification. Following population establishment in the laboratory, these species were reared on alternative hosts such as *Anticarsia gemmatalis* (Hübner, 1818) (Lepidoptera: Noctuidae) and *Tenebrio molitor* (Linnaeus, 1758) (Coleoptera: Tenebrionidae).

*Trichogramma pretiosum* was obtained from eggs of *Iridopsis panopla* (Prout, 1932) (Lepidoptera: Geometridae) in eucalyptus crops [26]. The eggs were maintained under controlled conditions (25 ± 2 °C, 70 ± 10% relative humidity, and a 14 h photophase) until parasitoid emergence. Specimens were sent to the “Oscar Monte” Entomophagous Insect Collection, located in Campinas, São Paulo, Brazil, at the Reference Laboratory Unit for Biological Control of the Biological Institute under the care of Dr. Nadja Nara Pereira da Silva. Subsequently, *Tg. pretiosum* were maintained in eggs of host *Ephestia kuehniella* (Zeller, 1879) (Lepidoptera: Pyralidae) [2].

### 2.2. DNA Extraction, Quantification and Sequencing

Each sample consisted of a pool of 50 adult individuals of the same species preserved in absolute ethanol and identified as PA03 (*P. elaeisis*), TH04 (*Te. howardi*), TD01 (*Ts. diatraeae*) and TP02 (*Tg. pretiosum*). Genomic DNA extraction was performed using the DNeasy Blood & Tissue Kit (Qiagen, Hilden, Germany), following the manufacturer’s protocol for insects [27]. Briefly, specimens were homogenized in 400 µL of lysis buffer, followed by the addition of 20 µL of Proteinase K, and incubated at 56 °C for 30 min to promote cell digestion. After incubation, samples were centrifuged at approximately 10,000× *g* for 1 min to pellet cellular debris. The supernatant containing genomic DNA was then transferred to a new tube and subjected to purification using a silica column system.

DNA quality was initially assessed by measuring the 260/280 absorbance ratio using a biophotometer. DNA concentration was quantified using the Quantus™ Fluorometer (Promega, Madison, WI, USA) together with the QuantiFluor ^®^ ONE dsDNA System reagent (Promega, Madison, WI, USA), following manufacturer’s recommendations.

Genomic libraries were prepared using the ABclonal Rapid Plus DNA Lib Prep Kit for Illumina V2 (ABclonal, Wuhan, China) and sequenced on the Illumina Novaseq 6000 platform (Illumina, San Diego, CA, USA), using the paired-read method (PE150), targeting an average sequencing depth of approximately 30× per sample. The choice of method was based on practicality for preparing libraries based on the selected genomic variants due to its high-throughput capacity and suitability for generating high-quality paired-end reads, enabling robust detection of genomic variation for comparative analyses among species, including those lacking reference genomes [28]. The resulting high-quality sequences were deposited in the GenBank Sequence Read Archive (SRA) under accession number: SRS24932171 for *P. elaeisis*, SRS24932172 for *Te. howardi*, SAMN48318921 for *Ts. diatraeae* and SRS24932169 for *Tg. pretiosum*.

### 2.3. Bioinformatic Analysis for Variant Detection

Raw sequencing read quality was assessed using FastQC (v0.11.8). Subsequently, reads were filtered and trimmed using fastp (v0.24.0) to remove adapters, poly-G tails associated with NovaSeq technology, and low-quality bases [29]. Reads shorter than 50 bp after filtering were discarded and the first 10 bases from the 5′ end of each read were trimmed to reduce potential sequencing biases. Sequencing summary statistics, including the number of reads and mean read length per sample before and after quality control, are presented in Table 1.

In order to identify the most suitable reference genome for maximizing species differentiation, a literature review was conducted to illustrate the phylogenetic proximity between the studied taxa and species with available genomic resources [30]. Based on this analysis, the four target species of this study (*Tetrastichus howardi*, *Palmistichus elaeisis*, *Trichospilus diatraeae*, and *Trichogramma pretiosum*) were aligned against three selected reference genomes: *Aphelinus certus* (Yasnosh, 1963) (Hymenoptera: Aphelinidae) and *Chouioia cunea* (Yang, 1989) (Hymenoptera: Eulophidae) and *Trichogramma pretiosum* (GenBank assembly accession GCA_000599845.3). Reference species were selected based on the availability of complete genomes in public databases and their relatively close phylogenetic relationship with the studied taxa. Sequences were aligned to identify the greatest number of common regions across the target species, considering only biallelic variants and excluding variants fixed among species, thereby enabling efficient genetic differentiation.

Filtered sequences were aligned to the selected reference genomes using the Burrows–Wheeler Aligner (BWA, v0.7.17) [31], allowing the identification of genomic regions shared among the studied species [16]. Alignment files were processed using BEDTools (v2.30.0) [32], allowing the identification of overlapping genomic intervals among the four target species for comparative genomic variant detection. Variant calling was performed using the mpileup algorithm implemented in SAMtools (v1.15) [33], retaining only biallelic single-nucleotide variants (SNVs). Variants were filtered according to the following criteria: sequencing depth greater than 10 reads (DP > 10), absence of multi-allelic sites, and removal of variants that were fixed between species or lacked at least 20 base pairs of high-quality flanking sequence on each side, ensuring sufficient genomic context for reliable downstream analyses. Only variants meeting these criteria were retained, ensuring their applicability for genetic discrimination among the studied taxa.

To validate the most suitable reference genome, a principal component analysis (PCA) was performed with two approaches: (i) using variants with a call rate above 0.5, and (ii) using variants present in the species of interest, enabling comparisons between the first principal component (PC1) observed in the sampling points of the following graphs. Subsequently, the selection of candidate variants was verified using the support vector machine (SVM) model for identification [31].

### 2.4. Principal Component Analysis, Marker Selection and Distance-Based Validation

Principal component analysis (PCA) was performed to explore the structure of the data and to identify highly informative markers. Two filtering strategies were applied: (i) variants with call rate > 0.5, and (ii) variants shared among all four species of interest and without missing data across samples. Considering the small sample size (n = 4), this threshold corresponds to variants genotyped in at least two samples, allowing retention of informative loci while minimizing data loss.

The PCA was conducted using the prcomp function in R [34], based on a matrix of allele proportions (reference allele dosage) that was centered and scaled. The analysis was used in an exploratory manner to identify markers with the highest contributions to PC1, PC2, and PC3, which were subsequently selected for downstream analyses aimed at species differentiation.

To quantitatively evaluate the discriminatory power of the selected markers, pairwise Euclidean distances were calculated from the same scaled matrix using the dist function in R version 4.4.2 [34]. The resulting distance matrix was visualized as a heatmap with hierarchical clustering using the pheatmap package [35]. Subsequently, candidate variants were further evaluated using the support vector machine (SVM) model for species classification [36], aiming to validate their discriminatory power among the studied taxa.

### 2.5. Sample Simulation

Considering the limited number of sequences available in the sample set of the target species, additional samples with similar genetic profiles were simulated to expand the test population and evaluate the robustness of variants for species differentiation in different scenarios. Sixty pure samples were simulated, with replications of the individual genetic profiles of *P. elaeisis*, *Te. howardi*, *Ts. diatraeae* and *Tg. pretiosum*. To evaluate the ability of variants to correctly identify different taxa, 18 mixed samples were generated, combining pairs of species in proportions of 25–75%, 50–50%, and 75–25%.

To validate the efficiency of the selected genetic variants, the data were subjected to PCA with two approaches: (i) using variants with a call rate above 0.5, and (ii) using variants present in the species of interest, allowing comparisons between the first principal component (PC1). Selected variants were validated in silico by the SVM model to predict species.

## 3. Results

### 3.1. Reference Genome

Genome sequencing generated high-quality data for the four parasitoid species analyzed (*P. elaeisis*, *Te. howardi*, *Ts. diatraeae*, and *Tg. pretiosum*), enabling the consistent identification of genomic variants in regions shared among taxa. The quality of the obtained sequences and the performance of the bioinformatic analysis allowed comparisons among the available reference genomes (Table S1).

The results indicate differences in performance among the evaluated reference genomes when considering variant detection and interspecies comparability (Table 2). Although *C. cunea* presented the highest total number of variants (n = 24,316) and *Tg. pretiosum* showed the highest number of common variants (n = 866), these results were also associated with a higher proportion of low-depth variants (DP < 10), which may affect the overall reliability of variant detection. Therefore, these metrics alone were not sufficient to ensure consistent discrimination among species.

In contrast, *A. certus* exhibited a more balanced performance across multiple criteria, including a lower number of fixed variants (n = 296) and a consistent proportion of high-quality variants, showing more consistent variant detection and alignment among allelic sequences across the analyzed species (Table 2).

The PCA, used here as an additional validation step, revealed clear differences among the evaluated reference genomes in terms of consistency and informativeness of variant detection. *Aphelinus certus* showed the highest concordance between the two filtering strategies, presenting the largest number of variants simultaneously identified in both approaches and shared across all four species (Table 2). Among the top-ranked variants contributing to PC1, 88 were common in the *A. certus* reference, compared to 82 for *C. cunea* and none for *Tg. pretiosum*, indicating greater robustness and reproducibility in the variants identified using *A. certus*.

Furthermore, *A. certus* exhibited the highest average length of shared genomic regions, suggesting improved continuity of aligned segments and a more reliable representation of polymorphic sites (Table 3). Overlaps as short as 1 bp may occur due to the intersection of regions across species. These results, together with the quality metrics described above, support the selection of *A. certus* as the most suitable reference genome for subsequent analyses.

### 3.2. Variant Identification and Differentiation

**Figure 1:**
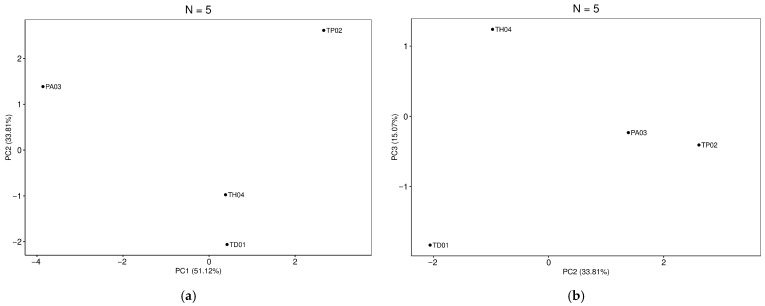
Principal component analysis (PCA) based on the most informative variants. (**a**) Projection of the samples in the space formed by the components PC1 (51.12%) and PC2 (33.81%), indicating the separation between the species. (**b**) Projection of the same samples in the PC2 (33.81%) and PC3 (15.07%) planes, reinforcing the consistency of the separation observed between the genetic groups.

PCA provided evidence for separation between species based on identified genomic variants. The first two principal components (PC1 and PC2) clearly separate the TP02 sample from the other samples with major variation along the first principal component which explains 51.12% of the total variation. From this separation, 15 candidate variants were identified as informative for species differentiation, of which 12 were able to perform this distinction in pairs, totaling 27 variants. In contrast, TD01 and TH04 appear closer, indicating a reduced genetic distance based on the evaluated variants. The second and third PC projections reinforce the differentiation among the samples, with TD01 showing a greater separation along the PC3, which explains 15.07% of the variation (Figure 1).

**Figure 2:**
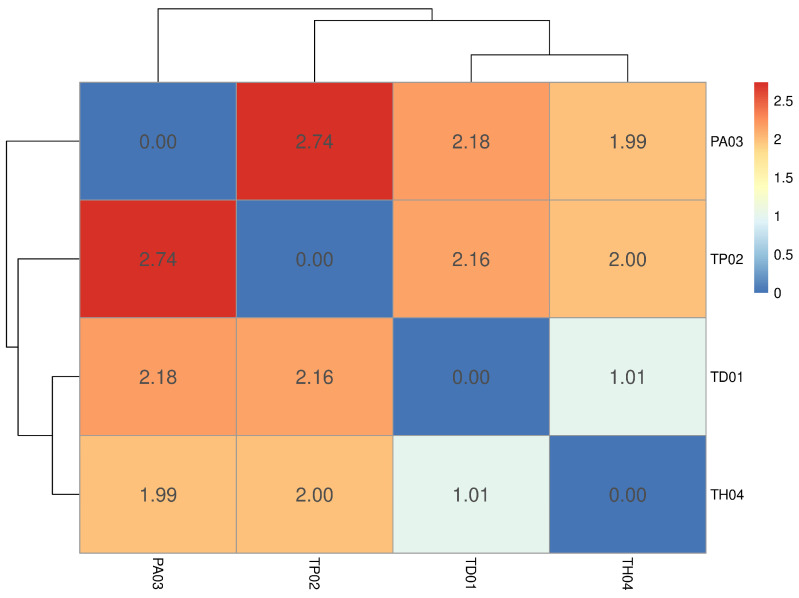
Heatmap of Euclidean distances among samples based on the selected set of informative genomic variants. Color intensity ranges from blue (lower genetic distance; 0) to red (higher genetic distance; 2.5), representing the magnitude of pairwise dissimilarity. Numerical values within each cell indicate the exact Euclidean distance between sample pairs.

This pattern is further supported by the Euclidean distance heatmap, which provides a quantitative assessment of genetic relationships among samples (Figure 2). The heatmap reveals shorter pairwise distances between TD01 and TH04, confirming their closer genetic relationship, while TP02 exhibits greater distances in relation to the other samples, consistent with the separation observed in the PCA. Overall, the Euclidean distance analysis reinforces the effectiveness of the selected variants in capturing interspecific genetic differentiation.

From this dataset, a subset of diagnostic variants was selected based on their contribution to species differentiation as identified through PCA and Euclidean distance analyses, as well as their allele frequency distribution among species. Table 4 summarizes SNPs showing species-specific patterns, in which the variant allele was fixed or highly frequent in one species while absent or rare in the others. Using this criterion, four diagnostic variants were identified for *P. elaeisis*, two for *Te. howardi*, four for *Ts. diatraeae* and five for *Tg. pretiosum* allowing their identification of each species individually. In addition to species-specific markers, a set of variants capable of differentiating species in pairwise comparisons was also identified (Table 5). These SNPs display contrasting allele frequencies between species pairs, allowing discrimination between combinations of taxa rather than a single exclusive lineage.

**Figure 3:**
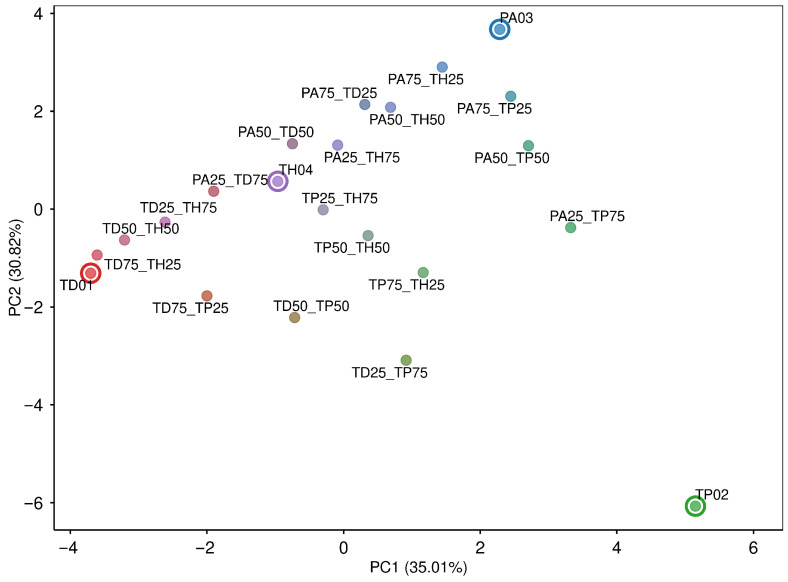
PCA based on simulations of mixed samples, evidencing the clear separation of pure samples and reinforcing the selection of informative variants.

An SVM model was implemented to test the efficiency of these variants in identifying the target species. The performance of the model was evaluated in both pure and mixed samples between multiple species, demonstrating high accuracy in the identification of the target species. The simulation of these species showed that mixed samples were positioned between their respective parental species, supporting the discriminatory capacity of the selected SNPs observed in the PCA (Figure 3).

Each variant was analyzed considering the 50 base pair flanking sequence on each side of the SNP (single-nucleotide polymorphism) type variant, allowing the construction of species-specific probes. This flanking length was defined based on standard criteria for primer and probe design, as regions of 18–25 bp free of variation are generally sufficient for primer binding [37], and the use of ~50 bp on each side ensures adequate sequence context for the selection of primers with suitable physicochemical properties and efficient amplification.

## 4. Discussion

Accurate species identification is essential for effective biological control. In this study, we established a molecular approach based on whole-genome sequencing to identify four parasitoid species *P. elaeisis*, *Te. howardi*, *Ts. diatraeae* and *Tg. pretiosum*. The identification of species-specific genomic variants provides a new resource for the reliable recognition of these biological control agents. In addition, the availability of genomic sequences in public databases such as GenBank enables future studies comparing populations from different regions, investigating phylogenetic relationships, and supporting systematic and comparative analyses [4].

An important methodological step in this study was the selection of reference genome for specific variant detection. Among the evaluated references, *A. certus* showed the best alignment performance, allowing the identification of informative genomic variants based on differences in nucleotide base calls that distinguished the target species. Although the use of a phylogenetically distant reference genome may reduce mapping efficiency and the number of variants detected, the generation of species-specific reference genomes still requires highly validated DNA sequences, advanced bioinformatics knowledge, and substantial financial investment [38]. Consequently, the use of reference genomes from related species has become a common strategy in genomic studies of non-model organisms when specific resources are not available [39]. Even under these conditions, informative variants can still be detected, preserving biologically significant patterns, such as the molecular discrimination of insects previously identified by morphological methods [40].

High-quality genomic sequencing technologies enable the generation of reference genomes and population datasets, contributing to biodiversity monitoring, conservation, and environmental restoration efforts [41]. In biological control programs, parasitoid populations are often multiplied for several generations in biofactories, which may lead to genetic changes associated with mass rearing [2]. Recent studies have shown that long-term mass rearing can reduce genetic diversity and alter population structure, highlighting the importance of genetic monitoring of biological control agents [42]. In the present study, the analysis of simulated mixed samples demonstrated the high accuracy of the selected genomic variants for species identification, even when one species represented only 25% of the sample and another species had 75% representation.

There is no doubt that DNA barcoding sequencing has become a standard and efficient genetic approach for species identification and biodiversity monitoring [43]. Over the years, molecular markers such as COI, ITS2, and 28S have been widely used in studies of parasitoid wasps [44,45,46]. Beyond their taxonomic applicability, these markers have also contributed to understanding processes such as speciation, gene flow, and local adaptation, particularly in ITS2 regions with discriminatory potential among closely related species [47,48]. In addition, markers such as COI and ITS2 have proven useful in quality control of mass rearing by enabling the identification of parasitoid lineages and the investigation of parasitoid–host interactions, including comparisons of genes encoding venom proteins between populations [49,50]. Together, these studies highlight the importance of molecular markers for distinguishing species at both intra- and interspecific levels.

However, sequencing small loci from informative DNA regions may limit the accurate characterization of genetic and taxonomic diversity within communities [41]. In this context, whole-genome sequencing allows the detection of species-specific variants, such as SNPs and indels, providing greater resolution for distinguishing closely related or morphologically similar taxa [16,21,51]. This approach also enables a more detailed investigation of the genetic bases of adaptive traits in insects, including mechanisms related to environmental adaptation and other evolutionary responses [52].

Furthermore, genomic datasets and publicly deposited sequences facilitate comparisons among lineages from different geographic regions and support the genetic monitoring of parasitoid populations used in biological control programs [49,53]. Thus, the advances obtained through whole-genomic sequencing and genetic analyses in this study contribute directly to strengthening the scientific bases of parasitoid production, promoting more efficient, reliable, and sustainable biological control programs.

## 5. Conclusions

The combination of high-quality DNA extraction and whole-genome sequencing enabled the identification of species-specific genomic variants for *P. elaeisis*, *Te. howardi*, *Ts. diatraeae* and *Tg. pretiosum*. The approach presented here establishes a novel genomic methodology for the precise molecular identification of these parasitoid species, including mixed samples. By providing reliable genetic markers and publicly available genomic data, these findings open new avenues for future research and have the potential to optimize the application of these species as biological control agents in sustainable agricultural and forestry systems.

## Figures and Tables

**Table 1:** Summary of sequencing reads and read length before and after quality control filtering.

Sample	Species	Number of Reads	Mean Read Length (bp)
BJ24000564-TH04	*Tetrastichus howardi*	31,074,280	150
BJ24000564-TH04_QC	*Tetrastichus howardi*	30,943,738	140
PA03	*Palmistichus elaeisis*	41,805,363	150
PA03_QC	*Palmistichus elaeisis*	41,693,209	140
TD01	*Trichospilus diatraeae*	43,207,578	150
TD01_QC	*Trichospilus diatraeae*	43,040,652	140
TP02	*Trichogramma pretiosum*	111,828,317	150
TP02_QC	*Trichogramma pretiosum*	111,157,289	140

**Table 2:** Selection of variants in the common regions of the target species *Palmistichus elaeisis*, *Tetrastichus howardi*, *Trichospilus diatraeae* and *Trichogramma pretiosum* with reference species *Aphelinus certus*, *Chouioia cunea* and *Tg. pretiosum* (Hymenoptera: Chalcidoidea). DP (Depth): depth of coverage. CR (Call rate): proportion of variants correctly identified.

Reference	Total	Indels and Multiallelic Systems	DP < 10	Quality	Fixes	Common Among the Four	CR > 0.5	TOP100
*Trichogramma pretiosum*	173,167	57,792 (33.34%)	95938 (55.35%)	18,164 (10.47%)	387 (0.22%)	866	4725	0
*Chouioia cunea*	192,976	25,543 (13.21%)	142,469 (73.71%)	24,316 (12.58%)	299 (0.15%)	349	5247	109 (82 + 27) ^1^
*Aphelinus certus*	121,009	17,355 (14.32%)	86,667 (71.52%)	16,162 (13.46%)	296 (0.24%)	529	3166	88 (88 + 0) ^1^

^1^ Sum of Major Components (PC1 + PC2) of common regions.

**Table 3:** Characterization of the number of overlapping genomic regions shared among *Palmistichus elaeisis*, *Tetrastichus howardi*, *Trichospilus diatraeae* and *Trichogramma pretiosum* (Hymenoptera: Chalcidoidea). Values correspond to the minimum, median, mean, and maximum lengths of these regions expressed in base pairs (bp).

Reference	Number of Regions	Minimum Length ^1^	Median Length ^1^	Mean Length ^1^	Maximum Length ^1^
*Trichogramma pretiosum*	244,745	1	16	19.23	934
*Chouioia cunea*	201,397	1	17	21.64	1934
*Aphelinus certus*	150,825	1	14	22.72	1132

^1^ Length of overlapping genomic regions.

**Table 4:** Specific variants of the target species *Palmistichus elaeisis* (PA03), *Tetrastichus howardi* (TH04), *Trichospilus diatraeae* (TD01) and *Trichogramma pretiosum* (TP02) (Hymenoptera: Chalcidoidea) that allow their individual identification.

	Freq					GQ *					
Variants	PA03	TD01	TP02	TH04	TH04	PA03	TD01	TP02	TH04	TH04	Specific
CM037083.1_8482984	1	0	1	1	1	127	127	127	127	117	TD01
CM037083.1_8483193	0	0	0	1	1	127	127	127	127	127	TH04
CM037083.1_8534743	0	0	0.004	1	1	127	127	127	127	127	TH04
CM037085.1_14072991	0.05	0.016	1	0	0	33	127	102	127	104	TP02
CM037087.1_13816	0	0.959	0.953	1	1	24	127	127	38	15	PA03
CM037087.1_13892	1	0.005	0.977	1	1	84	127	127	63	30	TD01
CM037087.1_3202	0.93	0.914	0	1	0.984	70	37	127	127	127	TP02
CM037087.1_3220	0	0.972	1	1	1	127	70	127	127	127	PA03
CM037087.1_8678	0.965	0	0.021	0	0	127	127	126	127	127	PA03
CM037087.1_8773	0.009	0.041	0.991	0	0	127	127	127	127	127	TP02
CM037087.1_8956	0	0.974	1	1	1	127	127	127	127	65	PA03
CM037087.1_9160	0.026	0	1	0	0	127	127	127	127	127	TP02
JAJGXB010002921.1_86	0.012	0	1	0	0	127	127	127	127	127	TP02
JAJGXB010011211.1_115	1	0	0.996	1	1	127	127	127	127	127	TD01
JAJGXB010011928.1_660	1	0	0.995	1	1	73	53	52	89	80	TD01

* GQ (Genotype Conditional Quality): Represents the quality of the genotypic call, encoded with Phred score and calculated by the formula (10log_10_ (p(genotype call is wrong|site is variant))); Higher GQ values indicate greater confidence in the accuracy of the assigned genotype.

**Table 5:** Variants for pairwise differentiation of the target species *Palmistichus elaeisis* (PA03)*, Tetrastichus howardi* (TH04), *Trichospilus diatraeae* (TD01) and *Trichogramma pretiosum* (TP02) (Hymenoptera: Chalcidoidea).

Variants	PA03	TD01	TP02	TH04	TH04	* GQ_PA03	GQ_TD01	GQ_TP02	GQ_TH04	GQ_TH04	* Low	* High
CM037083.1_8483200	1	0	0	1	0.987	127	127	127	127	127	TD01 × TP02	PA03 × TH04
CM037083.1_8483224	0	0.996	0	1	1	127	127	127	127	127	PA03 × TP02	TD01 × TH04
CM037085.1_14072988	0	0	1	1	1	121	127	112	127	76	PA03 × TD01	TP02 × TH04
CM037087.1_15154	0.991	0.992	0	0	0	127	127	127	127	127	TP02 × TH04	PA03 × TD01
CM037087.1_3238	0.994	1	0	0	0	127	127	127	127	127	TP02 × TH04	PA03 × TD01
CM037087.1_3241	0.006	1	0	1	1	127	127	127	127	127	PA03 × TP02	TD01 × TH04
CM037087.1_8968	0	1	1	0	0	127	127	127	127	76	PA03 × TH04	TD01 × TP02
CM037087.1_9106	1	0	1	0	0	127	127	127	127	127	TD01 × TH04	PA03 × TP02
CM037087.1_9133	0.005	0	1	1	1	127	127	127	127	127	PA03 × TD01	TP02 × TH04
JAJGXB010000273.1_8514	0	1	1	0	0	127	127	127	127	127	PA03 × TH04	TD01 × TP02
JAJGXB010011211.1_107	1	0	0	1	1	127	127	127	127	127	TD01 × TP02	PA03 × TH04
JAJGXB010012658.1_191	1	0	1	0	0	127	127	127	127	100	TD01 × TH04	PA03 × TP02

* GQ (Genotype Conditional Quality): Represents the quality of the genotypic call, encoded with Phred score and calculated by the formula (10log_10_ (p(genotype call is wrong|site is variant))); Higher GQ values indicate greater confidence in the accuracy of the assigned genotype. * Low: contains the species pairs with low frequency of the respective variant; High: shows the pairs with high frequency. Contributing to the differentiation between species.

## Data Availability

The original contributions presented in this study are included in the article/Supplementary Material. Further inquiries can be directed to the corresponding author.

## Supplementary Materials

The following supporting information can be downloaded at: https://www.mdpi.com/article/10.3390/insects17040395/s1, Table S1: Comparative sequencing and alignment metrics across reference genomes for the four parasitoid species.
